# Natural Compounds Modulate Drug Transporter Mediated Oral Cancer Treatment

**DOI:** 10.3390/biom10091335

**Published:** 2020-09-17

**Authors:** Hsiang Yang, Yu-Ching Wei, Wan-Chun Li, Hsin-Yung Chen, Hung-Ying Lin, Chun-Pin Chiang, Hsin-Ming Chen

**Affiliations:** 1 Department of Dentistry, National Taiwan University Hospital, College of Medicine, National Taiwan University, Taipei 100229, Taiwan; dtdent90@yahoo.com.tw (H.Y.); ephedrine0626@hotmail.com (H.-Y.L.); cpchiang@ntu.edu.tw (C.-P.C.); 2 Institute of Oral Biology, School of Dentistry, National Taiwan University, Taipei 100229, Taiwan; azure_0930@hotmail.com; 3 Institute of Oral Biology and Department of Dentistry, School of Dentistry, National Yang-Ming University, Taipei 11221, Taiwan; wcli@ym.edu.tw; 4 Cancer Progression Research Center, National Yang-Ming University, Taipei 11221, Taiwan; 5 Department of Occupational Therapy & Graduate Institute of Behavioral Sciences, College of Medicine, Chang Gung University, Taoyuan 333323, Taiwan; hychen@mail.cgu.edu.tw; 6 Department of Neurology and Dementia Center, Chang Gung Memorial Hospital, Taoyuan 333423, Taiwan; 7 Institute of Clinical Dentistry, School of Dentistry, National Taiwan University, Taipei 100229, Taiwan; 8 Department of Dentistry, Far Eastern Memorial Hospital, New Taipei City 220216, Taiwan

**Keywords:** ATP-binding cassette G2, oral cancer, photodynamic therapy, protoporphyrin IX, stemness

## Abstract

Oral cancer (OC) is a serious health problem. Surgery is the best method to treat the disease but might reduce the quality of life of patients. Photodynamic therapy (PDT) may enhance quality of life but with some limitations. Therefore, the development of a new strategy to facilitate PDT effectiveness has become crucial. ATP-binding cassette G2 (ABCG2) is a membrane protein-associated drug resistance and stemness in cancers. Here, we examined whether ABCG2 plays an important role in regulating the treatment efficacy of PDT and whether ABCG2 inhibition by natural compounds can promote the effect of PDT in OC cells. Several head and neck cancer cells were utilized in this study. OECM1 and SAS cells were selected to investigate the relationship between ABCG2 expression and protoporphyrin IX (PpIX) accumulation. Western blot analysis, flow cytometry analysis, and survival probability were performed to determine PDT efficacy and cellular stemness upon treatment of different dietary compounds, including epigallocatechin gallate (EGCG) and curcumin. In this study, we found that ABCG2 expression varied in OC cells. Hypoglycemic culture for SAS cells enhanced ABCG2 expression as higher ABCG2 expression was associated with lower PpIX accumulation and cellular stemness in OC cells. In contrast, suppression of ABCG2 expression by curcumin and tea polyphenol EGCG led to greater PpIX accumulation and enhanced PDT treatment efficiency in OC cells. In conclusion, ABCG2 plays an important role in regulating the effect of PDT. Change in glucose concentration and treatment with natural compounds modulated ABCG2 expression, resulting in altered PDT efficacy for OC cells. These modulations raise a potential new treatment strategy for early-stage OCs.

## 1. Introduction

Oral cancer (OC) is the sixth most prevalent cancer in the world [1]. The optimal treatment of oral cancer is radical surgery, which is combined with radiotherapy and/or chemotherapy in advanced cancer. However, surgical defects usually result in poor quality of life after operation. Among various alternative therapies, photodynamic therapy (PDT) is new for head and neck cancer (HNC) [2,3,4]. In our previous research, successful outcomes were reached by 5-aminolevulinic acid photodynamic therapy (ALA-PDT) for treatment of variant oral potentially malignant disorders [5,6,7,8,9,10,11] and oral cancer [12]. While topical ALA application showed a beneficial effect for treatment of oral mucosa lesions, the molecular cues to determine the treatment effect of ALA-PDT in oral premalignant or malignant lesions remains unknown.

ATP-binding cassette (ABC) is a family of membrane proteins. There are six subfamilies for ABC. ATP-binding cassette G2 (ABCG2) has been discovered in multidrug resistance cells [13]. ABCG2 functions as a transporter to export substrate drugs, including chemotherapeutic drugs, antibiotics, antivirals, and flavonoids, out of cells. Single nucleotide polymorphism of ABCG2 has been detected in various cancer cells and may contribute to a degree of resistance against chemotherapeutic drugs [13]. In agreement with its role in controlling treatment sensitivity, ABCG2 is also regarded as a marker to define stem cell population [14]. Previous studies have shown a selective accumulation of ALA-induced PpIX in oral potentially malignant disorders and malignant tissues, which is probably due to (1) limited capacity and/or low activity of ferrochelatase [15]; (2) differential PDT sensitivity in various cancer cell lines in combination with ABCG2 inhibition [16]. As ABCG2 has previously been shown to be critical for ALA-PDT in different human cancers, including brain and colorectal cancers [17,18,19], inhibition of ABCG2 could potentially enhance the efficacy of ALA-PDT in treatment of HNC.

As natural compounds are considered to be healthful and inexpensive compared to anticancer drugs, under research support, some natural compounds like curcumin and epigallocatechin gallate (EGCG) have been used as adjuvant agents for cancer prevention and treatment [20,21,22,23]. In this study, we aimed to delineate the importance of ABCG2 in regulating the effect of ALA-PDT treatment and investigate whether it is possible for natural compounds to suppress ABCG2 expression to enhance PDT treatment efficacy in OC cells.

## 2. Materials and Methods

### 2.1. Cell Lines and Culture

HNC cell lines, including OECM1, SAS, HSC3, and FaDu, were obtained from the Japanese Collection of Research Bioresources (Tokyo, Japan). Cancer cells were cultured in a medium depending on the requirement of varied survival condition of cells, which were previously described [24,25].

### 2.2. ALA-PDT

HNC cells were cultured in a six-well plate at a cell number of 8 × 10^5^ cells. HNC cells were pretreated with 1 mM ALA for 3 h as our previous study [26], then irradiated with red light (wave length: 635 ± 5 nm) at different doses of PDT depending on different experiment designs and cancer cells. The power density was 87mW/cm^2^, and the period of light treatment was set at 11.49 s to deliver an energy dose of 1 joule (J)/cm^2^. After ALA-PDT, those cancer cells were added to fresh medium and incubated for a further 24 h.

### 2.3. 3-(4, 5-Dimethylthiazol-2-yl)-2,5-diphenyltetrazolium bromide (MTT) Assay

MTT is a yellow tetrazole, which is reduced to purple formazan in a living cell. The half maximal inhibitory concentration (IC_50_) was used to detect the efficiency of ALA-PDT. A higher IC_50_ is considered to be lower efficacy of ALA-PDT.

### 2.4. Western Blot Analysis

The cells were collected and lysed to extract proteins, which were analyzed by following denaturation at 95 °C for 10 min and separation in 10% SDS-PAGE gel. Protein blot was then transferred to Polyvinylidene difluoride (PVDF) membrane and blocked with 5% nonfat milk in Tris-buffered saline with Tween-20 (TBST) at room temperature for 1 h to reduce background staining. The indicated primary antibody was applied at 4 °C for 16–20 h, and the membrane was incubated with horseradish peroxidase (HRP)-conjugated secondary antibody in TBST for 1 h at room temperature. The protein signals were presented with enhanced chemiluminescence (PerkinElmer, Waltham, MA, USA) and Fuji LAS-4000 lumino image analyzer (Fuji PhotoFilm, Tokyo, Japan). The primary antibodies included ABCG2, Nrf2 (Santa Cruz Biotechnology, Santa Cruz, CA, USA), p-EGFR (Abcam, Cambridge, UK), EGFR, PI3K, p-PI3K, Akt, p-Akt (Cell Signaling Technology, Beverly, MA, USA), β-actin, GADPH (GeneTex, Irvine, CA, USA). The secondary antibodies included anti-mouse IgG-conjugated HRP, anti-rabbit IgG-conjugated HRP (GeneTex, Irvine, CA, USA). Different inhibitors, including gefitinib, EGCG, curcumin, and LY294002 (PI3-Kinase Inhibitor), were purchased from Sigma-Aldrich (St. Louis, MO, USA).

### 2.5. PpIX Detection

The tested HNC cells were treated with ALA for 3 h. The concentrated cell pellets were extracted and tested by BD FACSCalibur™ flow cytometry (BD Bioscience, Franklin Lakes, New Jersey, USA) to detect the accumulation of PpIX with a FL3-H detection sensor.

### 2.6. Primary Tumor Sphere Culture

SAS cells were resuspended in defined serum-free medium consisting of serum-free DMEM/F-12, N2 supplement, 10 ng/mL human recombinant bFGF, and 10 ng/mL EGF as described previously [27].

### 2.7. ABCG2 Detection

Tested HNC cells were treated with Trypsin-EDTA to extract cell pellets. Those cell pellets were treated with ABCG2 antibody for 1 h to detect ABCG2 expression by BD FACSCalibur™ flow cytometry (BD Bioscience, Franklin Lakes, New Jersey, USA) with FL2-H detection sensor.

### 2.8. Aldehyde dehydrogenase (ALDH) Assay

ALDH activity was detected by ALDEFLUOR™ Kit (Stemcell technologies Inc., Vancouver, BC, Canada). The detected cells were added into ALDEFLUOR™ Assay Buffer as the test group, and the buffer contained BODIPY-aminoacetaldehyde (BAAA). ALDH^+^ cells can catalyze BAAA to be a fluorescent product, BODIPY-aminoacetate. Diethylaminobenzaldehyde (DEAB) is a specific inhibitor that is used as a negative control to define background ALDH activity. The assay was done by BD FACSCalibur™ flow cytometry (BD Bioscience, Franklin Lakes, New Jersey, USA).

### 2.9. Statistical Analysis

All data were analyzed using statistical software program package Prism 5 (GraphPad, San Diego, CA, USA) and SPSS 18.0 (SPSS Inc, Chicago, IL, USA). The multiple variant factors in groups were analyzed by a chi-square test, student’s *t*-test and one-way ANOVA analysis. A significant difference in the groups was defined as *p* value of less than 0.05.

## 3. Results

### 3.1. PpIX Accumulation Inversely Correlates with ABCG2 Expression

**Figure 1:**
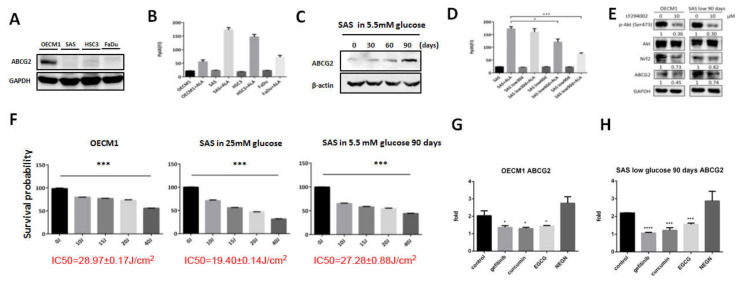
ATP-binding cassette G2 (ABCG2) expression was different in varied head and neck cancer (HNC) cell lines, which affected the amount of protoporphyrin IX (PpIX) and the survival percentage while the cells were treated with 5-aminolevulinic acid photodynamic therapy (ALA-PDT), and was modulated by different compounds. (**A**) Western blot analysis showed that ABCG2 was highly expressed in OECM1 but not in SAS, HSC3, and Fadu cells. (**B**) In parental status, PpIX amount of the four HNC cell lines showed no significant difference. For those cells treated with ALA, OECM1 produced the lowest amount of PpIX in comparison with SAS, HSC3, and Fadu cells. Switch of SAS cells from a medium containing 25 mM glucose to 5.5 mM glucose (**C**) induced ABCG2 expression and (**D**) downregulated PpIX accumulation in a time-dependent manner. (**E**) Western blot analysis for phospho-Akt, total Akt, Nrf2, and ABCG2 protein expression in OECM1 and SASL90d cells with/without treatment of PI3K inhibitor LY294002 (*n* ≥ 3; *p < 0.05; ***p < 0.001). IC_50_ of ALA-PDT treatment was measured in ABCG2-enriched cells: (**F**) OECM1, parental SAS, and SASL90d cells; (**G**) OECM1; and (**H**) SASL90d. ABCG2 level was significantly inhibited by gefitinib, epigallocatechin gallate (EGCG), and curcumin but not by naringenin. *n* ≥ 3, *p < 0.05, ***p < 0.001, ****p < 0.0001.

The association of ABCG2 expression and PpIX levels in different HNC cells was examined first. ABCG2 (Figure 1A) was differentially expressed in HNC cell lines, while PpIX levels were reversely correlated with ABCG2 expression in response to 1 mM ALA treatment (Figure 1B). Among all the tested cell lines, OECM1 cells expressed the most ABCG2 protein accompanied by the lowest level of intracellular PpIX. A previous study from our group showed that changes of glucose concentration in culture medium could modulate resistance to chemotherapeutic agents in HNC cells [25]. We therefore sought to further explore the potential role of ABCG2 in controlling glycemia-mediated drug sensitivity. While SAS cells were originally cultured in a medium containing 25 mM glucose, incubation of SAS cells in lower glucose concentration (5.5 mM) upregulated ABCG2 expression in a time-course manner (Figure 1C). Interestingly, PpIX accumulation was lower in SAS cells cultured in low glucose condition in a time-course manner (Figure 1D), suggesting a potential association between ABCG2 expression and intracellular PpIX levels in OC cells. To detect the pathway affecting ABCG2 expression in OC cells, treatment of PI3K inhibitor LY294002 downregulated ABCG2 and Nrf2 expression, suggesting that the Nrf2–ABCG2 signal could be a common molecular cue that controls PDT effectiveness in cancers (Figure 1E).

PDT efficacy was next examined in three different OC cells with differential ABCG2 expression levels. Cell viability of OECM1, SAS, and SAS cells cultivated in lower glucose condition (SASL90d) treated with different doses of ALA-PDT was recorded, and IC_50_ was used to determine treatment efficiency (Figure 1F). OECM1 cells exhibited the highest IC_50_ (28.97 ± 0.17 J) compared to the IC_50_ in SASL90d (27.28 ± 0.88 J) and parental SAS cells (19.40 ± 0.14 J), demonstrating that IC_50_ positively corresponds to ABCG2 expression.

### 3.2. Gefitinib, Curcumin, and EGCG Inhibited ABCG2 Expression and Modulated PpIX Accumulation and ALA-PDT Efficiency

**Figure 2:**
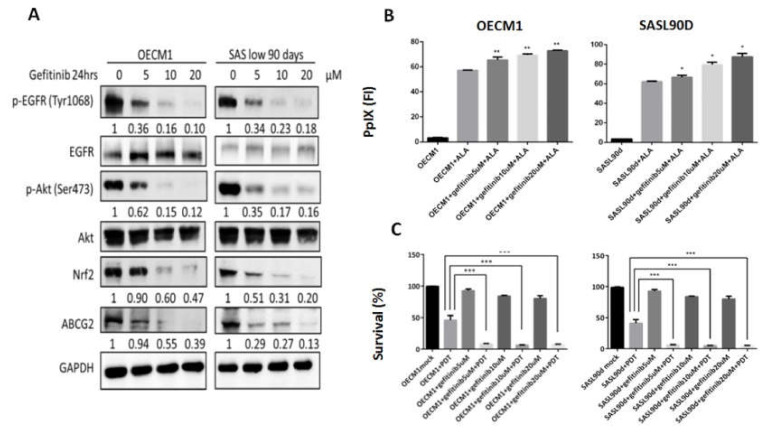
Gefitinib treatment suppresses ABCG2 expression, increases PpIx accumulation, and decreases ALA-PDT-mediated HNC cell viability. (**A**) Western blot analysis for p-EGFR (Y1068), p-Akt (S473), Nrf2, and ABCG2 expression in OECM1 and SASL90d cells treated with different concentrations of gefitinib. Gefitinib repressed EGFR and Akt signaling activities as well as ABCG2 and Nrf2 expression. In OECM1 and SASL90d, gefitinib treatment (**B**) upregulated PpIX accumulation and (**C**) decreased cell viability in a dose-dependent manner. *n* ≥ 3; *p < 0.05; **p < 0.01; ***p < 0.001.

**Figure 3:**
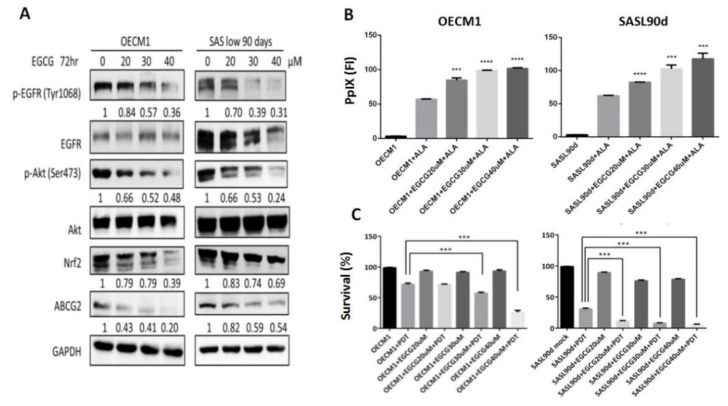
EGCG treatment suppresses ABCG2 expression, increases PpIx accumulation, and decreases ALA-PDT mediated-HNC cell viability. (**A**) Western blot analysis for p-EGFR (Y1068), p-Akt (S473), Nrf2, and ABCG2 expression in OECM1 and SASL90d cells treated with different concentrations of tea polyphenol EGCG. EGCG inhibited EGFR and Akt signaling activities as well as ABCG2 and Nrf2 expression. In OECM1 and SASL90d, EGCG treatment (**B**) upregulated PpIX accumulation and (**C**) decreased cell viability in a dose-dependent manner. *n* ≥ 3; ***p < 0.001, ****p < 0.0001.

**Figure 4:**
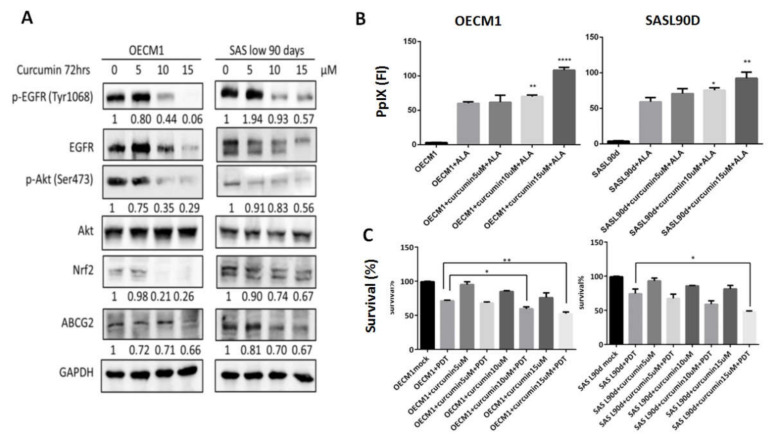
Curcumin treatment suppresses ABCG2 expression, increases PpIx accumulation, and decreases ALA-PDT-mediated HNC cell viability. (**A**) Western blot analysis for p-EGFR (Y1068), p-Akt (S473), Nrf2, and ABCG2 expression in OECM1 and SASL90d cells treated with different concentrations of curcumin. Curcumin inhibited EGFR and Akt signaling activities as well as ABCG2 and Nrf2 expression. In OECM1 and SASL90d, curcumin treatment (**B**) upregulated PpIX accumulation and (**C**) decreased cell viability in a dose-dependent manner. *n* ≥ 3; *p < 0.05; **p < 0.01, ****p < 0.0001.

As natural compounds can be potential anticancer agents, based on a number of studies, we further linked the effects of different anticancer compounds to how they control ABCG2 expression and ALA-PDT therapeutic sensitivity. To test the inhibitory effect, cells with greater ABCG2 expression, including OECM1 (Figure 1G) and SASL90d (Figure 1H) were selected for the experiment. The results showed that gefitinib, an EGFR inhibitor, curcumin, and tea polyphenol EGCG significantly inhibited ABCG2 levels compared with the untreated group. At the protein levels, ABCG2 protein expression was also inhibited by gefitinib (Figure 2A), EGCG (Figure 3A), and curcumin (Figure 4A) accompanied by suppression of p-EGFR (Tyr1068), p-Akt (Ser473), and Nrf2 in OECM1 and SASL90d cells.

The association of PpIX expression and the therapeutic effect of ALA-PDT was further examined. PpIX accumulation was increased in gefitinib-, EGCG-, and curcumin-treated OECM1 and SASL90d cells in a dose-dependent manner. (Figure 2B, Figure 3B, and Figure 4B, respectively). As for treatment efficacy in response to ALA-PDT under conditions with different treatments, the cell viability was inversely correlated with PpIX levels. Among various treatments, gefitinib (Figure 2C) and EGCG (Figure 3C) exhibited greater suppression compared with the effect of curcumin (Figure 4C).

### 3.3. ABCG2 Expression Correlated with Stemness, Tumor Grade, and Clinical Prognosis of HNCs

**Figure 5:**
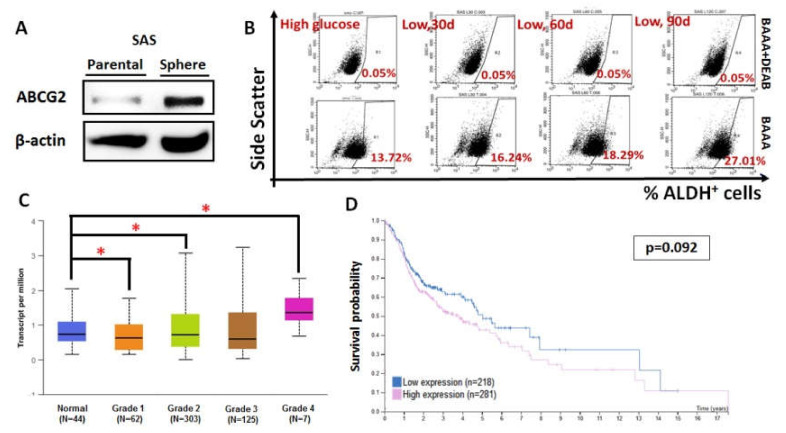
ABCG2 expression is associated with increased stemness, tumor grade, and HNC prognosis. (**A**) ABCG2 was enriched in SAS sphere culture compared with SAS parental cells. (**B**) Aldehyde dehydrogenase (ALDH)^+^ cell population gradually increased once SAS cells were cultured in a medium containing lower glucose. ABCG2 expression increased with increasing low-glucose culture time. ALDH^+^ percentage also increased with ABCG2 expression, with individual values of 16.24%, 18.29%, and 27.01%. BODIPY-aminoacetaldehyde (BAAA) is an ALDH substrate, and diethylaminobenzaldehyde (DEAB) is an inhibitor of ALDH activity. (**C**) Statistical analysis for ABCG2 mRNA levels in normal and primary HNC tissues stratified by clinical grades from UALCAN database. *p < 0.05. (**D**) Kaplan–Meier analysis for cancer-specific survival rates in HNSCC patients classified by ABCG2 expression using the Human Protein Atlas database. The expression cut-off value is 0.32 FPKM (fragments per kilobase of transcript per million mapped reads).

Previous studies have established a sphere culture system to enrich cancer stem cell-like population from oral squamous cell carcinoma (OSCC) [27]. To define the association of ABCG2 expression and cancer stemness in HNC cells, we determined ABCG2 protein expression in a SAS sphere culture system. It was found that ABCG2 protein was upregulated in SAS sphere culture compared to parental two-dimensional culture (Figure 5A), indicating that ABCG2 acts as a potential stemness marker in HNC cells. It was also found that parental SAS cells incubated in lower glucose exhibited higher ALDH^+^ cell population (Figure 5B), in agreement with the fact that ABCG2 expression was upregulated in SASL90d compared to parental SAS cells (Figure 1C). The clinical impacts of ABCG2 in HNCs was further analyzed using the Cancer Genomic Atlas (TCGA)-based UALCAN [28] and the Human Protein Atlas database [29,30]. ABCG2 transcript in HNCs was highly expressed in undifferentiated (grade 4) tumor tissues in contrast to normal head and neck tissues (Figure 5C), while ABCG2 levels negatively correlated with patient prognosis (Figure 5D), implying that ABCG2 could serve as a prognostic marker for HNCs in clinics.

## 4. Discussion

The current study showed that ABCG2 expression varies in HNC cells derived from different origins (oral cavity cancer cells: OECM1, HSC3, and SAS; hypopharyngeal cancer cell: FaDu). In agreement with a previous study [31], it was also shown that ABCG2 expression in HNC cells could be inhibited by the EGFR inhibitor gefitinib and natural compounds curcumin and EGCG, thereby possibly modulating ALA-PDT therapeutic efficacy. The potential association of ABCG2 expression and cell stemness, HNC differentiation status, and clinical outcomes provides an important insight on the role of ABCG2 in controlling HNC cell malignancy.

ABCG2 is a membrane protein and functions to pump endogenous or exogenous compounds out of cells, including PpIX or chemotherapeutic agents [13]. This membrane protein is not only presented in cell membrane but also in mitochondrial membrane, acting as a functional transporter of PpIX to determine the effect of ALA-PDT [32]. In the present study, we performed glycemic switch from a medium containing 25 mM glucose to lower concentration (5 mM) in SAS cells and found that ABCG2 expression was increased and a lower amount of intracellular PpIX was accumulated, thereby resulting in a higher survival rate. Consistent with our data, Cheng and To also found that glucose deprivation upregulated ABCG2 expression [33]. At the molecular level, a previous study demonstrated that the Nrf2–ABCG2 signaling axis might be involved in the intrinsic resistance to methyl pyropheophorbide-amediated photodynamic therapy in human ovarian cancer cells [34]. ABCG2 expression in SASL90d could be suppressed by the Akt inhibitor LY294002, possibly through Nrf2 inhibition. Taken together, our results demonstrate that environmental glucose levels could modulate cellular ABCG2 expression and the ability of PpIX export and sequential PDT efficiency.

One of the most well-known roles of ABCG2 is that ABCG2 expression is closely related to drug resistance and stemness [35,36]. Sphere culture is considered to enrich stem-like cell population. Our data showed that ABCG2 expression was higher in SAS spheres than SAS parental cells, supporting the earlier suggested role of ABCG2 protein as a stemness marker. A very recent study by Sasaki et al., however, showed that spheres derived from ABCG2^−^ pancreatic cells displayed higher stemness markers and exhibited greater sphere formation capacity than ABCG2^+^ cells [37], leaving the origin of ABCG2^+^ cells in question.

Photodynamic therapy is an inexpensive but time-consuming therapy. In our previous experience of treating verrucous carcinoma [12], we successfully treated stage 4 cases with ALA-PDT. However, it is noteworthy that PDT does exhibit shortcomings in a clinical setting as PDT is only effective in treating early-stage OC patients due to limiting penetration depth of the light used. Thus, even though a good outcome was observed in our clinical treatment scheme with ALA-PDT, a long-term therapeutic procedure taking almost a year to complete treatment for particular tumorous lesion still makes PDT efficacy an issue for clinical intervention; complete treatment for that particular tumorous lesion still took almost one year. Therefore, how to increase PDT efficiency has become a very important issue to address. As ABCG2 expression determines the PpIX level in cells, taking advantage of suppressing ABCG2 expression and thereby promoting PDT efficiency could be feasible. Based on a former study by Ishikawa et al. [38], we used gefitinib, curcumin, and EGCG to inhibit ABCG2 expression in HNC cells. As gefitinib, EGCG, and curcumin inhibited ABCG2 expression, although to varying degrees, reducing intracellular PpIX accumulation and increasing oral cancer cell viability, these results suggest that there might be a possible benefit of treating early-stage oral cancer patients with PDT in combination with EGCG or curcumin to enhance treatment effectiveness in a future medication plan.

## 5. Conclusions

Overall, in this study, we found that ABCG2 could affect ALA-PDT therapeutic efficiency in OC cells. The underlying pathways for ABCG2-mediated regulation could possibly be via an EGFR-related route. On the other hand, EGCG and curcumin had similar effects to gefitinib in suppressing ABCG2 expression. High ABCG2 expression corresponded to HNC stemness, tumor grade, and clinical outcome, showing that ABCG2 is important in controlling ALA-PDT efficacy to treat HNC. Therefore, using the natural compounds curcumin and EGCG to suppress ABCG2 expression in combination with PDT is a potential strategy to treat oral cancer.
